# Review of Bacterial Nanocellulose as Suitable Substrate for Conformable and Flexible Organic Light-Emitting Diodes

**DOI:** 10.3390/polym15030479

**Published:** 2023-01-17

**Authors:** Thales Alves Faraco, Marina de Lima Fontes, Rafaella Takehara Paschoalin, Amanda Maria Claro, Isabella Salgado Gonçalves, Mauricio Cavicchioli, Renan Lira de Farias, Marco Cremona, Sidney José Lima Ribeiro, Hernane da Silva Barud, Cristiano Legnani

**Affiliations:** 1 Laboratory of Organic Electronics (LEO), Department of Physics, Federal University of Juiz de Fora (UFJF), Juiz de Fora 36036-330, MG, Brazil; 2 Laboratory of Molecular Optoelectronics (LOEM), Department of Physics, Pontifical Catholic University of Rio de Janeiro (PUC-Rio), Rio de Janeiro 22451-900, RJ, Brazil; 3 Laboratory of Biopolymers and Biomaterials (BioPolMat), Laboratory of Medicinal Chemistry and Biomaterials (LQMBio), Department of Biotechnology, University of Araraquara (UNIARA), Araraquara 14801-340, SP, Brazil; 4 Center of Exact Sciences and Technology, Federal University of São Carlos (UFSCar), São Carlos 13565-905, SP, Brazil; 5 Department of Chemistry, Pontifical Catholic University of Rio de Janeiro (PUC-Rio), Rio de Janeiro 22451-900, RJ, Brazil; 6 Laboratory of Photonic Materials, Department of Analytical, Physical-Chemistry and Inorganic Chemistry, Institute of Chemistry, State University of São Paulo (UNESP), Araraquara 14800-060, SP, Brazil

**Keywords:** OLED, FOLED, BNC, bacterial nanocellulose

## Abstract

As the development of nanotechnology progresses, organic electronics have gained momentum in recent years, and the production and rapid development of electronic devices based on organic semiconductors, such as organic light-emitting diodes (OLEDs), organic photovoltaic cells (OPVs), and organic field effect transistors (OFETs), among others, have excelled. Their uses extend to the fabrication of intelligent screens for televisions and portable devices, due to their flexibility and versatility. Lately, great efforts have been reported in the literature to use them in the biomedical field, such as in photodynamic therapy. In tandem, there has been considerable interest in the design of advanced materials originating from natural sources. Bacterial nanocellulose (BNC) is a natural polymer synthesized by many microorganisms, notably by non-pathogenic strains of *Komagataeibacter* (*K. xylinus*, *K. hansenii*, and *K. rhaeticus*). BNC shows distinct physical and mechanical properties, including its insolubility, rapid biodegradability, tensile strength, elasticity, durability, and nontoxic and nonallergenic features, which make BNC ideal for many areas, including active and intelligent food packaging, sensors, water remediation, drug delivery, wound healing, and as conformable/flexible substrates for application in organic electronics. Here, we review BNC production methods, properties, and applications, focusing on electronic devices, especially OLEDs and flexible OLEDs (FOLEDs). Furthermore, we discuss the future progress of BNC-based flexible substrate nanocomposites.

## 1. Introduction

A new type of electronics, organic electronics, has emerged with the development of new organic semiconductor materials. The academic and industrial interest in the development of electronic devices based on organic substrates is due to the ease in processing the devices, lower cost, control over optoelectronic properties, flexibility, elasticity, and possible interface with living tissues.

Organic semiconductor materials are applied in organic light-emitting diodes (OLEDs), organic field effect transistors (OFETs), organic thin film transistors (OTFTs), and organic photovoltaic devices (OPVs). Among the different technologies, the production of OLEDs has many advantages over current display technology, such as high efficiency, high luminous intensity, and low operating voltage. These devices are made of heterostructures consisting of a substrate on which thin layers of organic material are deposited between two electrodes. Among the structures, the substrate, although it is only the support of the active device, has a fundamental role in the construction of flexible OLED devices. In this context, biocompatible and biodegradable materials represent the perfect solution for the design of biocompatible devices that can be integrated and interfaced with biological systems.

In this work, we provide a short overview of bacterial nanocellulose as a substrate for flexible organic light-emitting diode production.

## 2. Bacterial Nanocellulose: Versatile Material

Cellulose (C_6_H_10_O_5_)_n_ has received considerable and growing attention in the electronic/electrochemical/magnetic field, as well as in food packaging, the cosmetic area, bioengineering, and reinforcement materials to make blends, composites, and nanocomposites [1]. It is the most abundant biopolymer produced in the biosphere, composed of D-glucose monomers connected by β (1–4) glycosidic bonds [1,2,3]. It can be synthesized by plants, animals, and microorganisms, notably by non-pathogenic bacteria belonging to the genus *Komagataeibacter* (*K. xylinus, K. hansenii,* and *K. rhaeticus*) [2,3,4].

**Figure 1:**
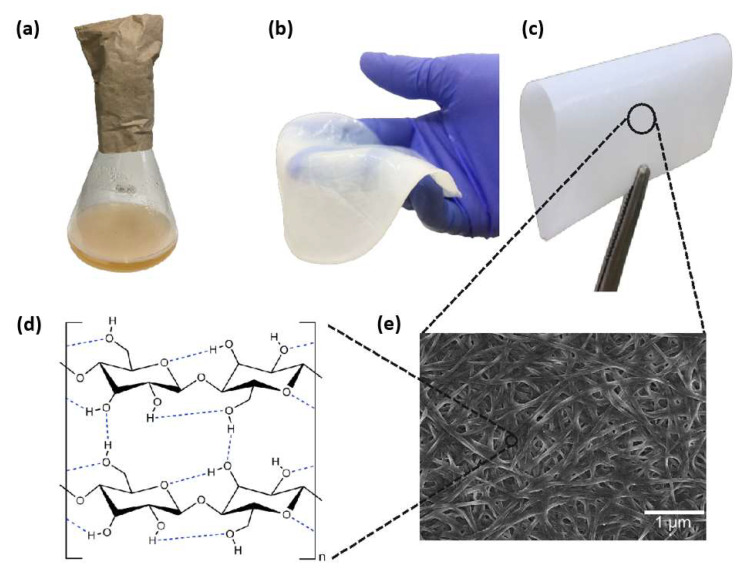
BNC (**a**) hydrogel obtained in static culture media, (**b**) purified hydrogel, (**c**) dry flexible membrane, (**d**) chemical structure, and (**e**) scanning electron microscopy image showing a typical tridimensional nanofibrous network.

Bacteria cultivated in a culture media rich in carbon, nitrogen, and mineral sources secrete cellulose in the form of 3D web-shaped microfibrils, also known as bacterial cellulose, or bacterial nanocellulose (BNC). The production of BNC gelatinous pellicles occurs through traditional static cultivation. The production time and carbon sources control the BNC hydrogel thickness (25–65 μm) [4,5]. Despite that, none of the above techniques manages thicknesses under 1 μm. Figure 1 shows the BNC pellicle in the wet and dry states and the nanometric fibrillar structure formed from cellulose chains.

Both 3D-arranged fiber networks in nanometric dimensions (diameter ca. 30 nm) and the high number of classical H-bonds on cellulose structures allow the unique properties of BNC [2,6], such as high crystallinity (89%) [7], high tensile strength (200–300 MPa), and high Young’s modulus (up to 78 GPa) [8]. Moreover, the insolubility of BNC in common solvents [6], its high thermal stability, high flexibility, lightweight, low thermal expansion coefficient, and negligible light scattering make it a promising substrate for replacing glass on traditional rigid OLEDs [8]. BNC possesses much higher crystallinity, liquid absorption capacity, and mechanical properties when compared to plant nanocellulose. Furthermore, the possibility of producing BNC thin films in submicron dimensions (under 1 μm) [9] is quite attractive. Liu F. et al. [10] report a straightforward method for the biosynthesis of ultrathin BNC mats, in which the thickness was varied by controlling the depth of the culture broth so that films with predictable thicknesses, between 0.113 and 1.10 μm, were obtained.

Substrates based on BNC have been widely employed during optoelectronic and electronic device development. Usually, these substrates are designed as BNC pellicles, BNC membranes, BNC biocomposites, or composites.

## 3. Organic Light-Emitting Diodes (OLEDs)

The first OLED operating at low voltage (<10 V) was developed in 1987 by Ching W. Tang and Steven Van Slyke [10]. Three years later, Jeremy H. Burroughes and collaborators from the University of Cambridge reported for the first time an efficient OLED based on a conjugated polymer [11]. OLED is a light-emitting diode (LED) [12,13] in which the layers between the electrodes (anode and cathode) are made of organic semiconductors. At least one of the electrodes must be transparent, which is where the light will emerge. The architecture of OLEDs is formed by one or more layers with thicknesses ranging from a few to several nanometers. They are interleaved as uniform thin films one on top of the other forming a heterojunction.

OLEDs usually exhibit high luminous efficiency, brightness, fast response time, low operating voltage, and low power consumption, and they are relatively simple to manufacture in small and large areas. In addition, their light color emission can be tuned upon an appropriate choice of their architecture and organic molecules [14,15]. Commonly, a multilayer OLED is produced by the following organic layers: the hole injector layer (HIL), the hole transporting layer (CTB), the emissive layer (EML), the electron transporting layer (ETL), and the electron injector layer (EIL). Furthermore, to achieve more efficiency, it is not uncommon to find OLEDs with both hole-blocking (CBB) and electron-blocking (CBE) layers. The emissive layer is selected to emit light at the required wavelength, and the other layers are used to optimize the performance of the device [16,17].

**Figure 2:**
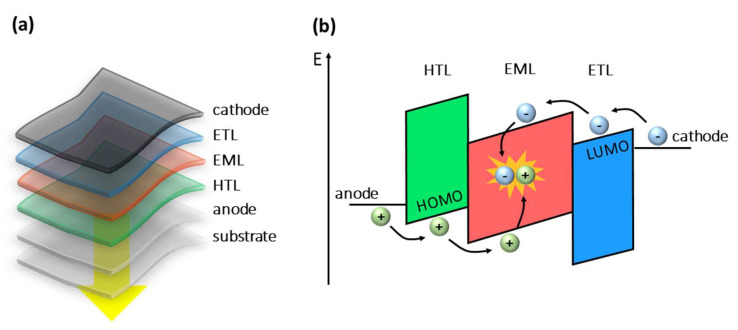
(**a**) Structure of an OLED with three organic layers (electron transport—ETL, hole transport—HTL, and emissive—EML). (**b**) Schematic representation of the HOMO−LUMO energy levels of different materials, where the OLED processes in operation are shown: (1) injection, (2) transport, (3) recombination (formation of excitons), and (4) emission.

OLEDs work by converting electrical energy into light. The main mechanisms of electroluminescence involve four main steps, as shown in Figure 2: (1) charge injection, (2) charge transport, (3) charge recombination, and (4) light emission. When a voltage (external electric field) is applied between the electrodes, holes (positive carriers) and electrons (negative carriers) are injected from the anode and cathode, respectively. They are conducted through the organic layers, where electrons are transported through the lowest unoccupied molecular orbital (LUMO) and holes through the highest occupied molecular orbital (HOMO), moving in opposite directions towards the recombination zone (emissive layer) for the formation of electron–hole pairs (excitons). This recombination results in addition to non-radiative decays, in the emission of visible light through radiative decays. The carriers are transported mainly by a hopping mechanism within the HOMO and LUMO, which must have enough energy to overcome the barriers at the interfaces between the materials. The energy barriers have a triangular shape due to the effect of the applied electric field [16,17].

OLEDs are typically classified based on the nature of the EML (small-molecule organic light-emitting diodes—SMOLEDs [18] and polymer-OLEDs—PLEDs [19]); on the direction of light emission including bottom-emitting OLEDs [20] and top-emitting OLEDs [21]; on the transparency (transparent OLEDs—TOLEDs [22]); on the display structure (passive-matrix OLEDs—PMOLEDs [23] and active-matrix OLEDs—AMOLEDs [24]); and based on the mechanical flexibility—FOLEDs [25]. In addition, OLEDs that emit white light are called WOLEDs [26].

OLEDs have been traditionally fabricated on rigid glass sheet substrates. Glass sheets are the most used type of substrate due to their excellent physical and chemical properties, such as transparency in the visible region and thermal stability. However, they have the disadvantage of not being able to be flexed or bent, which limits their applicability in flexible devices. To use new flexible substrates and make them good conductors to be used in the construction of FOLEDs [27], some fundamental requirements must be satisfied: for instance, they must have the most homogeneous and smooth surface possible and have good chemical, thermal, and mechanical resistance. All this is necessary since high roughness and irregularities on the substrate surface can impair the electrical properties of the films and the manufacturing and operation steps of FOLEDs. Finally, the substrates must be transparent in the visible region to be used in the manufacture of devices in which light is emitted through the substrates (such as bottom-emitting OLEDs and TOLEDs).

Transparent conducting oxide (TCO) thin films, such as tin-doped In_2_O_3_ (ITO), ZnO, and aluminum-doped zinc oxide (AZO) [28], have been used extensively for several years in the production of conductive substrates due to their high electrical conductivity and high transparency in the visible region. However, nanostructured materials, such as silver, gold nanowires [29,30,31], and reduced graphene oxide [32], have been considered ideal materials to replace the existing ones and make the substrates more flexible.

Biopolymeric sustainable materials exhibit the requirements mentioned above [33,34,35]. Moreover, biopolymers are biodegradable (which contributes to the reduction in environmental electronic pollution) and are biocompatible, which provides potential applications in the biomedical field. Substrates based on BNC [36,37,38], silk fibroin [39,40,41], *Allium cepa* L. [42], and gellan gum [43] have been reported in the literature for the construction of organic optoelectronic devices. In these works, the use of deposition techniques by thermal evaporation, sputtering, electron beam, and spin-coating has been very recurrent. The first three involve physical processes (PVD—physical vapor deposition) and the last involve chemical processes (CSD—chemical solvent deposition). All of these techniques produce uniform, high-quality thin films for building efficient FOLED devices [44].

### 3.1. BNC-Based Substrates for FOLED Applications

All works listed chronologically below refer to the use of BNC substrates or BNC-based composites for the manufacture of flexible organic light-emitting diodes (FOLEDs). In general, they refer to substrates as environmentally friendly, biocompatible, conformable, and/or flexible, and suggest applications in the technology sector for the manufacture of flexible displays, as well as in the biomedical field to be used in photodynamic therapy (PDT), for example.

In 2008, Legnani et al. [45] reported the preparation of FOLEDs using BNC membranes with 40% optical transmittance at 550 nm. To make the BNC conductive, ITO thin films 185 nm thick were grown on them by rf magnetron sputtering using 30 W of power and 8 mPa of pressure in an argon atmosphere. The depositions were performed at room temperature and without subsequent heat treatment. In some BNC, before the ITO, a silicon dioxide interlayer (90 nm SiO_2_) was deposited directly on the membrane surface using 50 W and 6 mPa. They report the need for this SiO_2_ interlayer to make the deposition of ITO films more homogeneous and uniform (by reducing BNC surface roughness), to prevent diffusion or possible chemical reactions on the BNC surface and to avoid micro-cracks. They reported that the SiO_2_ film reduced the surface roughness of the BNC substrate from 120 nm to 45 nm using the atomic force microscopy (AFM) technique, which corresponds to a reduction of more than 60%. ITO films were deposited on glass, SiO_2_/BNC, and BNC substrates.

**Figure 3:**
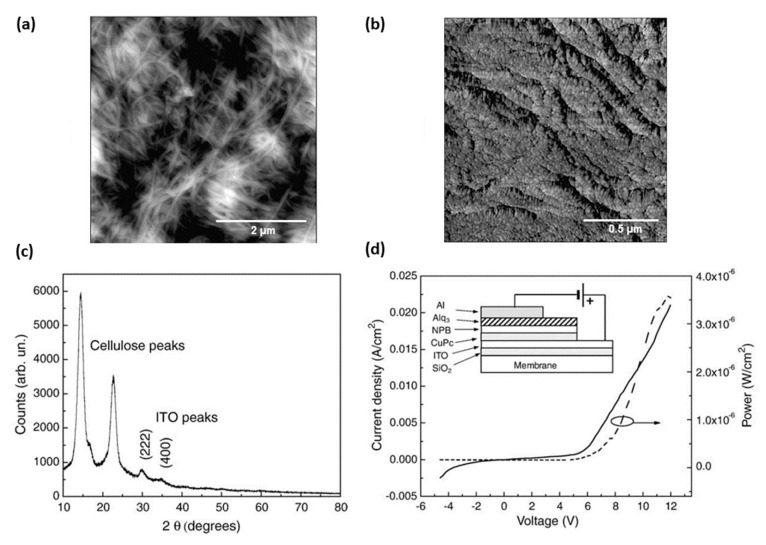
AFM images of the surface of the (**a**) BNC membrane with the presence of microfibrils and of the (**b**) ITO/SiO_2_/BNC substrate where ≈20 nm ITO grains were observed. (**c**) X−ray diffraction pattern of the ITO/BNC substrate showing the cellulose and ITO peaks. (**d**) Current and power density curves for FOLED manufactured on ITO/SiO_2_/BNC substrate. The insert shows the architecture of the FOLED on the BNC substrate. Reproduced with permission from ref. [45]. Copyright 2022, Elsevier, *Thin Solid Films*.

The ITO/SiO_2_/BNC substrate showed a resistivity of 4.9 × 10^−4^ Ω cm against 9.0 × 10^−4^ Ω cm exhibited by the BNC substrate without the SiO_2_ interlayer (ITO/BNC), i.e., a higher electrical conductivity. Therefore, all ITO films showed electrical resistivity in the same order of magnitude as commercial ITO film (10^−4^ Ω cm), as shown summarized in Table 1. ITO films showed grain size estimated to be approximately 20 nm (Figure 3b), and revealed a cubic bixbyte structure with (222) and (400) orientation perpendicular to the substrate (Figure 3c) [45].

Furthermore, three OLEDs were produced: (i) a reference device using commercial ITO/glass substrate, (ii) an ITO/SiO_2_/BNC device, and (iii) an ITO/BNC device (without the SiO_2_ interlayer). The organic and aluminum films were deposited by thermal evaporation in a high vacuum environment (1 mPa) with deposition rates of 0.2 and 1.0 nm/min, respectively. The active area of the devices was about 5 mm^2^ and the architecture was as follows: conductive substrate/15 nm CuPc/45 nm NPB/50 nm Alq_3_/120 nm Al. Measurements were performed without device encapsulation, that is, at ambient atmosphere. The OLEDs were operated under forward bias voltage, with the ITO acting as the anode and aluminum as the cathode. The maximum luminance values obtained were about 2400 cd/m^2^ for the reference OLED and 1200 cd/m^2^ for the FOLED on ITO/SiO_2_/BNC substrate (Figure 3d). The better performance shown by the reference OLED was justified due to that the pinholes can be more frequent in BNC, leading to a higher incidence of short circuits and the consequent destruction of the device. FOLED manufactured on ITO/BNC substrate revealed half of the maximum luminance displayed by the FOLED manufactured with a SiO_2_ interlayer (600 cd/m^2^). The SiO_2_ film, in addition to making the surface less rough, reduces the appearance of micro-cracks throughout the ITO films, improving injection and charge conduction in the devices [45].

Nanocomposites composed of BNC and polyurethane (PU) based resin, were investigated as flexible and transparent substrates to be used in FOLEDs by Ummartyotin, S. et al. in 2012 [46]. The substrates exhibited high optical transmittance, with values ranging from 75–87% in the visible region (400–800 nm). BNC-PU nanocomposites were thermally stable up to 150 °C, with a degradation temperature of 345 °C. 

**Figure 4:**
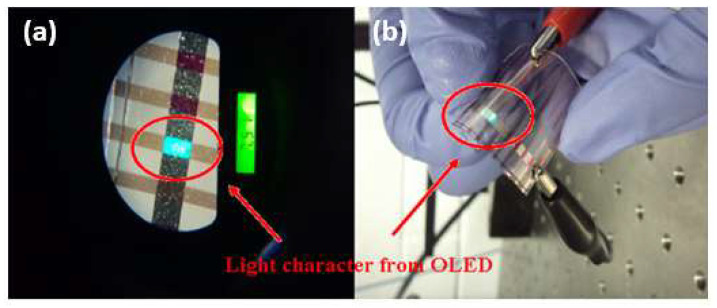
Digital photographs of the FOLED in operation manufactured on the nanocomposite when it was (**a**) flat and (**b**) bent. Reproduced with permission from ref. [46]. Copyright 2022, Elsevier, *Industrial Crops and Products*.

They manufactured a FOLED on the BNC-PU substrate using the following architecture: BNC-PU/200 nm Cu/1.5 nm MoO_3_/50 nm CBP/50 nm Alq_3_/1 nm LiF/100 nm Al. The Cu, MoO_3_, and Al films were deposited in a separate metallization chamber with a base pressure of about 1.4 × 10^−5^ Pa. The MoO_3_ film was treated by ex situ oxidation with the UV ozone for 30 min. The organic layers and LiF film were deposited by thermal evaporation in a high vacuum environment (~1.4 × 10^−6^ Pa). The FOLED was operated under forward bias voltage, with the Cu electrode acting as the anode and the Al electrode acting as the cathode. The FOLED showed a turn-on voltage at 12 V, which according to the authors was comparable to OLED made on a commercial ITO-coated glass substrate. In addition, a small operating voltage is desired to avoid OLED destruction. They attributed the good performance displayed by the device to the uniform thickness of the layer. The FOLED exhibited maximum current efficiency of 0.085 cd/A and maximum power efficiency of 0.021 lm/W at maximum luminance of 200 cd/m^2^. The authors qualitatively demonstrated, through digital photographs, that the manufactured FOLED could emit light when it was flat (Figure 4a) and bent (Figure 4b) [46].

**Figure 5:**
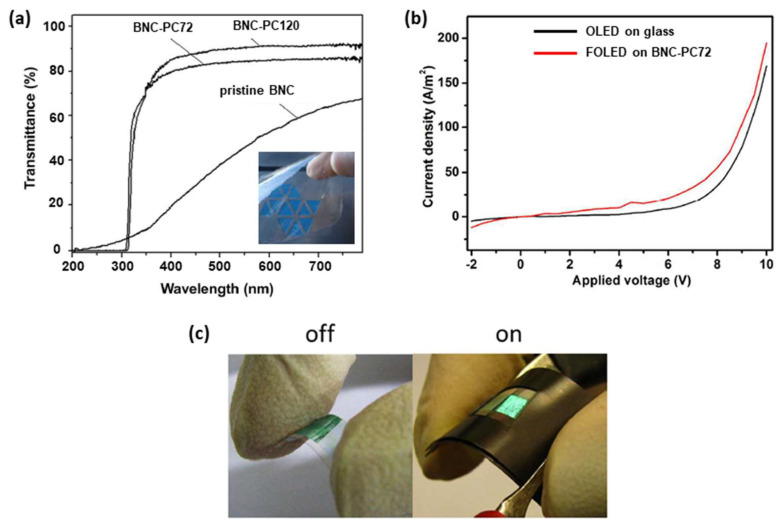
(**a**) Optical transmittance spectra comparing the transparency of BNC-PU composites with the pristine BNC. The inset shows a digital image of the BNC-PU72 substrate. (**b**) Current density curves for OLED manufactured on BNC-PU72, and on ITO-coated glass substrates. (**c**) Digital photographs of the FOLED on BNC-PU composite in “on” and “off” states. Reproduced with permission from ref. [47]. Copyright 2022, Royal Society of Chemistry, *Journal of Materials Chemistry*.

Likewise, Pinto et al. in 2015 [47] proposed flexible and transparent composites prepared from BNC and castor-oil-based polyurethane (PU) to be used in FOLEDs. They examined the optical transmittance spectra of the pristine BNC and two composites, which were obtained using a solvent exchange process for PU incorporation in 72 h (BNC-PU72) and 120 h (BNC-PU120). The authors found that composites showed a significant increase in transmittance compared with pristine BNC. For example, at 350 nm, the increase was over 62%. The BNC-PU72 and BNC-PU120 substrates showed a high transmittance at 700 nm of about 82% and 90%, respectively (Figure 5a). They reported a very low roughness of less than 1 nm for BNC-PU composites and 32 nm for pristine BNC. BNC-PU composites showed good thermal stability (>250 °C) and excellent mechanical properties, with tensile strength up to 69 MPa and Young’s modulus up to 6 GPa.

A SiO_2_ thin film (100 nm) and ITO thin films (300 nm) were deposited on BNC-PU72 substrate by rf magnetron sputtering using powers of 100 W and 80 W, respectively. ITO films were also deposited on glass substrate to act as a control group. All the depositions were performed with a pressure of 0.72 Pa. The ITO-coated BNC-PU72 substrate showed an electrical resistivity of 5.78 × 10^−4^ Ω cm, which is a value very close to that exhibited by the same film deposited on glass substrate (3.29 × 10^−4^ Ω cm). They attributed the difference in electrical resistivity value mainly to the flexible characteristics of the BNC-PU substrate (see Table 1) [47].

Two OLEDs were produced: (i) a reference device using an ITO-coated glass substrate and (ii) a device based on ITO/SiO_2_/BNC-PU72. The OLEDs were manufactured by thermal evaporation in a high vacuum environment (1 mPa) with the following architecture: conductive substrate/CuPc/NPB/CBP co-deposited with Ir(ppy)_3_/BCP/Alq_3_/Al. Deposition rate values were 3 nm/min for organics and 18 nm/min for aluminum. The devices fabricated on the ITO/SiO_2_/BNC-PU72 substrate showed a maximum luminance of 231 ± 18 cd/m^2^ versus the 485 ± 8 cd/m^2^ exhibited by the reference OLED (Figure 5b). Technical difficulties in BNC-PU OLED measurement were reported due to folds during the data acquisition process, which may have impaired the luminance measurement. However, the FOLED on BNC-PU presented a lower applied voltage and a higher current density value, revealing a better performance. Digital photographs of the FOLED made on the BNC-PU composite in the on/off states were also presented (Figure 5c) [47].

**Figure 6:**
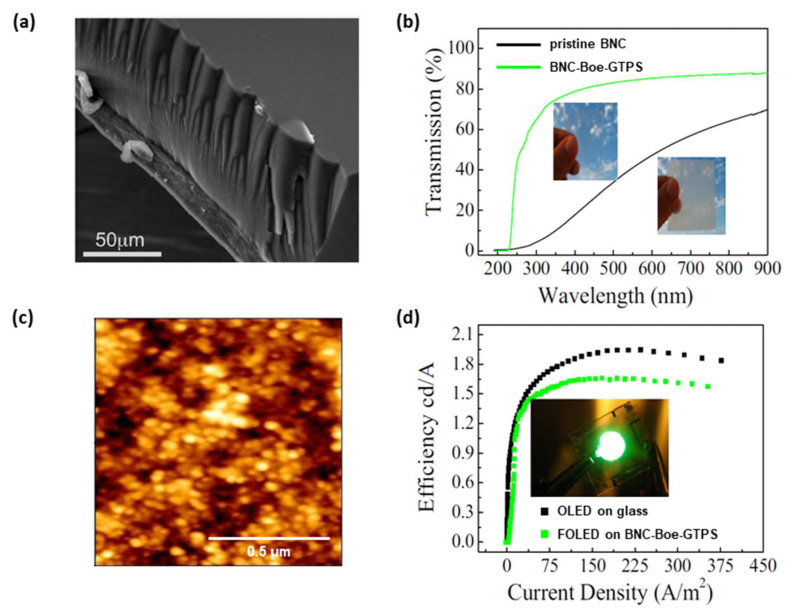
(**a**) SEM micrograph represents a cross section of the BNC-Boe-GTPS nanocomposite: Boe-GTPS (top); BNC (bottom). (**b**) Optical transmittance spectra and digital images comparing the transparency of the nanocomposite with the pristine BNC. (**c**) AFM images of the surface of the ITO/SiO_2_/BNC-Boe-GTPS substrate with the presence of ≈30 nm ITO grains. (**d**) Current efficiency curves for FOLED on ITO/SiO_2_/BNC-Boe-GTPS and commercial ITO-coated glass substrates. The digital image of the FOLED manufactured on the nanocomposite is shown in the inset. Reproduced with permission from ref. [48]. Copyright 2022, Springer Nature, *Journal of Materials Science: Materials in Electronics*.

In 2019, Legnani et al. [48] reported a considerable improvement in the performance of FOLEDs previously manufactured on pristine BNC [45]. In this current study, the authors investigated the development of nanocomposites produced using BNC and an organic-inorganic sol, composed of boehmite (Boe) nanoparticles and epoxy-modified siloxane (GTPS) (Figure 6a). The nanocomposite showed optical transmittances of 70% at 300 nm and 88% at 700 nm, respectively. It was found that the transparency of nanocomposites increased considerably to 88% at 550 nm when compared to the 40% achieved for the pristine BNC reported in previous work (Figure 6b).

ITO thin films (300 nm) and a SiO_2_ interlayer (90 nm) (between the ITO and the nanocomposite) were deposited by rf magnetron sputtering on BNC-Boe-GTPS substrate at room temperature and without subsequent heat treatment. The SiO_2_ and ITO films were grown in an argon atmosphere at 50 W and 6 mPa for the SiO_2_ and at 60 W and 200 mPa for the ITO. After the deposition of SiO_2_ and ITO films, a surface roughness of 10 nm was reported using the atomic force microscopy (AFM) technique, which represents a value 42 times smaller than that of the surface of the BNC-Boe-GTPS before the deposition of thin films [48].

The ITO/SiO_2_/BNC-Boe-GTPS substrate showed an electrical resistivity of 2.7 × 10^−4^ Ω cm, which is a value very close to that exhibited by the commercial ITO substrate in glass (2.1 × 10^−4^ Ω cm), as shown summarized in Table 1. The surface of the ITO film was observed with an approximate 30 nm grain size, which may indicate that the films were polycrystalline (Figure 6c) [48].

The authors fabricated two Alq_3_-based OLEDs by thermal evaporation with the same architecture and active area reported in a previous work [45]: a reference device using commercial ITO on glass and another device on ITO/SiO_2_/BNC-Boe-GTPS (FOLED). The depositions were performed in a high vacuum environment using a base pressure of 1 mPa. The organic layers and aluminum film were evaporated at a rate of 0.2 and 1.0 nm/min, respectively. OLED measurements were performed in a glove box nitrogen atmosphere and without device encapsulation, with ITO and aluminum films as positive and negative electrodes, respectively. They achieved maximum current efficiency of 1.95 cd/A for the reference OLED and 1.68 cd/A for the FOLED on ITO/SiO_2_/BNC-Boe-GTPS substrate (Figure 6d). In addition, they reported a 50% increase in device current efficiency over the same OLED on pristine BNC published in a previous work [48].

**Figure 7:**
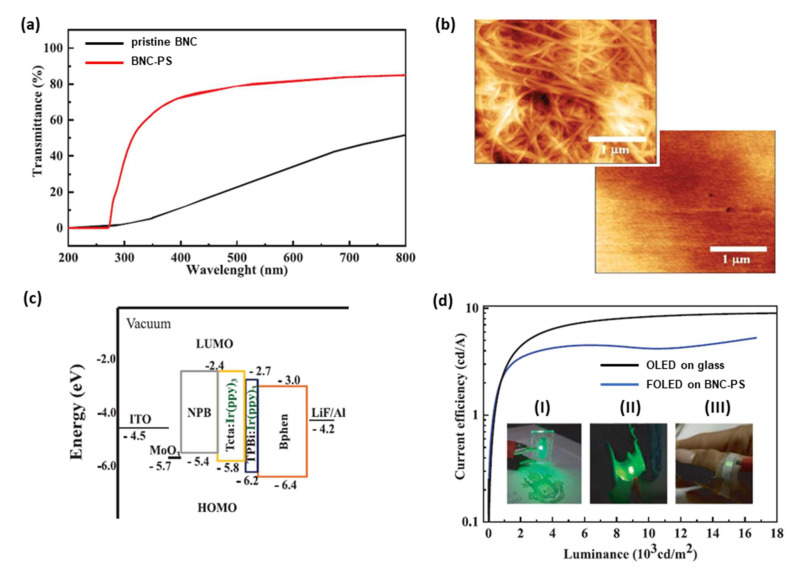
(**a**) Optical transmittance spectra comparing the transparency of BNC-PS composite with the pristine BNC. (**b**) AFM images of the pristine BNC surface (**top**) and BNC-PS composite (**bottom**), demonstrating lower surface roughness of BNC-PS. (**c**) Energy level diagram of OLEDs manufactured. (**d**) Current efficiency curves for FOLED on BNC-PS and commercial ITO−coated glass substrates. The inset presents digital photographs of the (I) OLED reference, (II) FOLED on the BNC-PS substrate, and (III) an illustration of a potential application of the BNC-PS OLED in Photodynamic Therapy. Reproduced with permission from ref. [49]. Copyright 2022, John Wiley and Sons, *Advanced Sustainable Systems*.

Recently (2022), Cebrian et al. [49] developed composites based on BNC modified with recycled polystyrene (PS) to be used in FOLEDs. The substrates became more transparent when higher PS concentrations from 0 to 15% were used. However, BNC-PS substrate (10%) showed better uniformity and optical transmittance of 83% at 550 nm. This value was much higher in comparison to the pristine BNC, which exhibited optical transmittance of 37% (Figure 7a).

The BNC-PS roughness was 4 nm, which expresses a reduction of more than 89% of the value exhibited by pristine BNC (39 nm), as can be seen in Figure 7b. BNC-PS composites showed good thermal stability up to 270 °C and excellent mechanical properties, with tensile strength of 189 ± 59 MPa and Young’s modulus of 2.6 ± 0.8 GPa, which was a higher conformability than commercial PET (1.7 GPa). Furthermore, 200 nm of the SiO_2_ interlayer was deposited by rf magnetron sputtering on BNC-PS substrate to reduce surface roughness. Subsequently, to make the substrates conductive ITO films were deposited on SiO_2_/BNC-PS using 20 W power and 0.34 Pa pressure for 40 min in an argon atmosphere [49].

The authors reported a nominal sheet resistance of approximately 20 Ω/sq for the ITO/SiO_2_/BNC-PS substrate. Following that, the organic molecules and aluminum film were sequentially deposited by thermal evaporation in a high vacuum environment (1.4 × 10^−4^ Pa). Two OLEDs were produced: a reference device using commercial ITO-coated glass and another device on ITO/SiO_2_/BNC-PS substrate [49].

The structure of the OLEDs consisted of: conductive substrate/2 nm MoO_3_/30 nm β-NPB/20 nm TcTa co-deposited with Ir(ppy)_3_ (10%)/10 nm TPBi co-deposited with Ir(ppy)_3_ (10%)/30 nm Bphen/0.1 nm LiF/100 nm Al (Figure 7c). The active area of the OLED was 3 mm^2^ and the organic layers were evaporated at a rate of 0.5 A/s. Measurements were performed in ambient atmosphere and temperature without OLED encapsulation. All devices exhibited a turn-on voltage of approximately 3.0 V. The maximum luminance, current efficiency and power density shown by the BNC-PS OLED were 16,000 cd/m^2^, 5 cd/A at and 2.6 mW/cm^2^, respectively (Figure 7d). According to the authors, the results of the BNC-PS substrate were comparable to standard glass, although the commercial ITO-coated glass substrate demonstrated better performance [49].

## 4. BNC as a Template for Other Devices

BNC has emerged as an attractive material for the development of eco-sustainable electrodes and on-skin electronic patches capable of detecting and monitoring different biofluids such as glucose, alcohol, cholesterol, etc. [50]. Conductive polymers, metal nanoparticles, and carbon-based materials are some materials that have been used in the preparation of novel platforms using BNC as a template.

Polyaniline (PAni), a conductive polymer, was incorporated into the BNC network through the oxidative polymerization reaction using ammonium persulfate as an oxidant in association with different dopants. The conductivity reached the maximum 5.0 × 10^−2^ S/cm when the reaction time was 90 min. Higher conductivity was confirmed when PANI was doped with HCl. The authors concluded that the nanocomposites demonstrated excellent mechanical properties combined with conductive properties derived from PAni [51].

Jasim et al. [52] also reported BNC-PAni film fabrication in association with single-walled carbon nanotubes (SWCNTs) that have shown considerable electrical conductivity and environmental stability. The authors confirmed a gradual increase in conductivity after the impregnation of PAni and SWCNTs, reaching an electrical conductivity current of 4.64 × 10^−3^ S/cm for BC-PAni/SWCNTs. Moreover, the linear sweep voltammetry technique was used to investigate the use of BC-PAni/SWCNTs film nanocomposites as a working electrode, demonstrating the higher electroactive potential when SWCNTs were impregnated with BC-PAni. Thus, BNC-PAni and BC-PAni/SWCNT composites could be used in different applications, including flexible electrodes, biosensors, and display devices.

Khan et al. [53] investigated the fabrication of nanocomposites composed of AuNPs (gold nanoparticles) and ink of PEDOT:PSS onto a BNC substrate. The nanocomposite films showed an electrical conductivity between 2.95 × 10^−2^ S/cm and 16.65 ± 1.27 S/cm attributed to AuNPs and extended conjugation with PEDOT:PSS, respectively. Additionally, the BNC-AuNPs-PEDOT:PSS nanocomposites did not show a toxic effect, proving their high potential for biosensor fabrication directed for medical diagnosis.

Metal-organic frameworks (MOFs) were used as sacrificial agents to fabricate a flexible supercapacitor based on BNC. This device demonstrated excellent capacitance (around 59.8% at 20 mA cm^−2^) and good cycling stability, opening a novel platform for research on next-generation energy devices. Other studies have demonstrated the use of BNC hydrogel as a solid electrolyte for the fabrication of flexible and safe supercapacitors [54].

**Figure 8:**
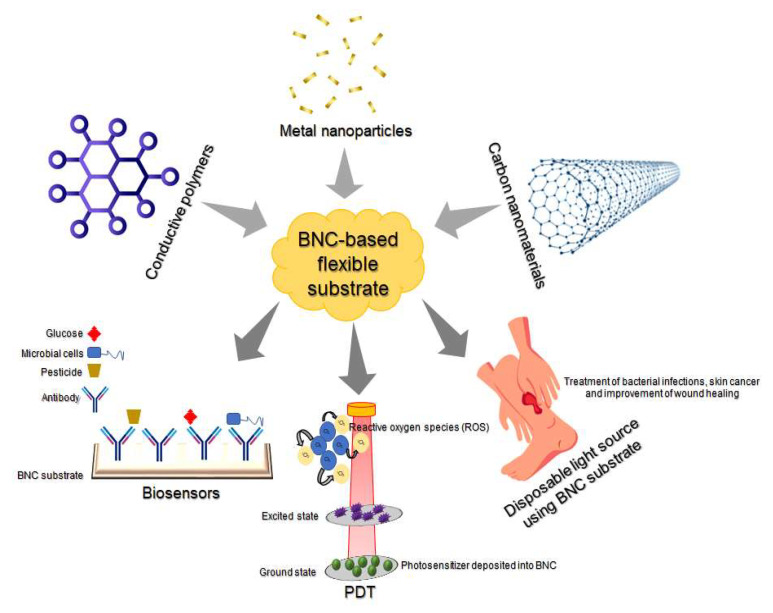
Main applications of BNC-based flexible substrate in the biomedical area.

Due to their great ability to absorb exudates and attach to irregular skin surfaces, BNC films were also evaluated as flexible substrates loaded with a photosensitizer named chloroaluminum phthalocyanine (ClAlPc) and luminescent upconversion nanoparticles, respectively, for photodynamic therapy (PDT) [55]. The BNC-ClAlPc films showed higher luminescence intensity, longer lifetime based on luminescence-decay kinetics, and toxicity when tested against Chinese hamster ovary cells (CHO-K1). Therefore, this study proved the feasibility of BCN as a substrate to incorporate photosensitizers and its vast application in the treatment of skin carcinoma, skin infections, and mucosal disease. In this context, substrates based on BNC emerge as a great alternative for PDT, and with the potential to be scaled up to a large area could be applied in treatment of superficial bacterial infections and to improve the wound healing process [56]. All these applications are demonstrated in Figure 8.

## 5. Discussion

BNC has attracted much attention over the last twenty years as a functional biomaterial for optoelectronic devices due to its lightweight, transparency, flexibility, and high surface area [57]. In terms of composition, bacterial cellulose is a polymer structurally similar to plant cellulose. However, it has improved physicochemical properties due to the well-organized three-dimensional network of fibers with diameters ranging from 3.0 to 3.5 μm. These, in turn, are assembled by bundles of thinner cellulosic fibers with diameters ranging from micro- to nanoscale [58].

In this review, we focused on studies that used BNC as a substrate for FOLED development and discuss their results. As we observed, one of the most relevant parameters is the surface morphology of the substrates, which in its pure form presents a relatively high roughness, ca. 120 nm, impairing the proper functioning of the OLED. In this sense, post-treatment to dewater the matrices exhibits the most significant influence on the resulting morphology of pure BNC hydrogels. However, efforts have been made to functionalize the dried BNC films, aiming to smooth the surface before applying them as an OLED substrate. Deposition of the silicon dioxide layer (ca. 90 nm) by magnetron sputtering is a successful example of smoothing the surface of BNC. In addition, this thin layer allows the deposition of uniform, homogeneous, transparent, and conductive ITO films, maintaining their optoelectronic properties. It is important to emphasize that this strategy avoids the appearance of micro-cracks and prevents diffusion or some chemical reaction at the BC–film interface.

Surface roughness also influences light scattering and consequently limits the optical properties of BNC. The optical transmittance of pure BNC is incipient (≈37%) in the visible range of the electromagnetic spectrum, being necessary to increase the transmittance to improve the total light emitted by the OLED. Accordingly, a strategy to increase BNC transmittance is filling the interstitial voids in the three-dimensional structure of the nanofibers, thus reducing light scattering. For this, the dopant must have a light refractive index similar to that found for the BNC (η = 1.5). BNC has been used to develop other smart devices based on biocomposites, including biosensors and photosensitive films [59]. Although not the focus of this work, herein we briefly report the current research, and some of its technological applications.

The intense interaction between hydroxyl groups in BNC fibers leads to a tendency for self-assembly. As a result, an extended network is observed via classical intra- and intermolecular hydrogen bonds, allowing the production of mats with high surface area and porosity. Furthermore, the mechanical properties, such as the tensile strength (ca. 300 mPa) and Young’s modulus (ca. 80 GPa) [58], make this a valuable biopolymer for other technological applications. In addition, as far as the biomedical area is concerned, the most attractive property of BNC is its biocompatibility and non-cytotoxic activity. Therefore, since its discovery, it has proved to be a suitable platform, such as in artificial skin, artificial blood vessels, microvessels, wound dressings, second- or third-degree implants, and dental implants [60].

## 6. Conclusions

Concerns about sustainability have attracted interest in renewable materials from nature as emerging solutions to a range of technological challenges. In particular, cellulose-based materials are not only earth-abundant and biocompatible but also have nature-provided intrinsic properties for potentially transformative impacts on new recyclable electronic and photonic devices, such as paper-based energy devices, solar cells, transistors, smart packaging, and point-of-care (PoC) devices, among others.

The performance of OLEDs strongly depends on the controlled structure of the BNC produced. There is a direct relationship between the structure, porosity, dimensions, and surface properties of the BNC and its behaviors. Accordingly, controlling these characteristics is crucial for obtaining the advantage of using BNC and BNC composites in high-performance energy storage devices.

In this work, we provide a short overview of bacterial nanocellulose as a substrate for flexible or conformable OLED production. Generally, these devices based on BNC composites showed lower roughness, high optical transmittance, and high luminance, in addition to higher electrical resistivity when compared to a commercial ITO-coated glass substrate. Additionally, substrates based on BNC emerge as a great alternative for PDT, flexible electrodes, and biosensors.

## Figures and Tables

**Table 1:** Summary of the optoelectronic properties of the conductive and transparent BNC substrates and the manufactured OLEDs.

Optoelectronic Properties									
Conductive Substrates	Thickness nm		T % (550 nm) *	ρ 10^−4^ Ω cm	n 10^20^ cm^−3^	µ - cm^2^ V^−1^ s^−1^	OLED		Ref.
	SiO_2_	ITO					Maximum Luminance cd m^−2^	Current Efficiency cd A^−1^	
ITO/SiO_2_/BNC	90	185	40	4.9	−5.0	8.1	1200	-	[45]
ITO/BNC	-	185	40	9.0	−5.8	6.5	600	-	[45]
Comm. ITO/glass (Asahi)	-	≈120	-	2.4	−20.0	23.4	2400	-	[45]
ITO/glass	-	185	-	5.0	−8.3	13.7	-	-	[45]
Cu/PU-BNC	-	-	90	-	-	-	200	0.085	[46]
ITO/SiO_2_/BNC-PU72	100	300	85	5.78	−5.17	20.89	231 ± 18	-	[47]
ITO/glass	-	300	-	3.29	−9.55	19.89	485 ± 8	-	[47]
ITO/SiO_2_/BNC-Boe-GTPS	90	300	88	2.7	−14.8	15.2	-	1.68	[48]
ITO/BNC	-	300	40	3.0	−21.5	9.67	-	0.84	[48]
Comm. ITO/glass (Lumtec)	-	≈120	-	2.1	−20.0	23.44	-	1.95	[48]
ITO/SiO_2_/BNC-PS	200	-	83	R_s_: 20 Ω/sq	-	-	16,000	5	[49]
Comm. ITO/glass	-	≈120	-	-	-	-	18,000	9	[49]

T: optical transmittance; ρ: electrical resistivity; R_s_: sheet resistance; n: carrier concentration; µ: carrier mobility. * Transmittance of bacterial cellulose-based substrates before film deposition.

## Data Availability

Not applicable.
